# A6 INVESTIGATING THE RELATIONSHIP BETWEEN MUCOSAL INFLAMMATION AND PROTEOLYTIC ACTIVITY IN CROHN’S DISEASE

**DOI:** 10.1093/jcag/gwae059.006

**Published:** 2025-02-10

**Authors:** J Szeto, G H Rueda, A Hann, M Constante, X Wang, C Deraison, N Vergnolle, J Butcher, A Stintzi, P Bercik, H Galipeau, D Armstrong, E Verdu

**Affiliations:** Medicine, McMaster University Faculty of Health Sciences, Hamilton, ON, Canada; Medicine, McMaster University Faculty of Health Sciences, Hamilton, ON, Canada; Medicine, McMaster University Faculty of Health Sciences, Hamilton, ON, Canada; Medicine, McMaster University Faculty of Health Sciences, Hamilton, ON, Canada; Medicine, McMaster University Faculty of Health Sciences, Hamilton, ON, Canada; INSERM, Toulouse, France; INSERM, Toulouse, France; University of Ottawa Faculty of Medicine, Ottawa, ON, Canada; University of Ottawa Faculty of Medicine, Ottawa, ON, Canada; Medicine, McMaster University Faculty of Health Sciences, Hamilton, ON, Canada; Medicine, McMaster University Faculty of Health Sciences, Hamilton, ON, Canada; Medicine, McMaster University Faculty of Health Sciences, Hamilton, ON, Canada; Medicine, McMaster University Faculty of Health Sciences, Hamilton, ON, Canada

## Abstract

**Background:**

Dysregulated proteolytic activity (PA) has been detected in intestinal biopsy samples of individuals with inflammatory bowel disease (IBD) compared to healthy controls. It is known that increased microbial fecal PA precedes ulcerative colitis (UC) diagnosis in an at-risk population. Intestinal inflammation in UC is characterized by continuous ulceration, inflammation in Crohn’s disease (CD) is patchy in nature. Sites of inflammation in CD has been associated with altered mucosa-associated microbes and higher permeability, suggesting these could be localized sites of increased microbial PA.

**Aims:**

To investigate whether inflamed mucosal areas in CD are associated with proteolytic microbiota and increased permeability.

**Methods:**

CD patients in flare (n=23) were recruited at McMaster University Medical Centre and matched (or paired) biopsies were collected at colonoscopy from inflamed and adjacent (within 5 cm) non-inflamed sites. Biopsy mucosal-to-serosal ^51^Cr-EDTA flux rate and tissue conductance were measured with Ussing chambers. Total proteolytic (trypsin), elastase-like (FITC-elastin), and mucolytic (0.05% mucin plates) activities were also assessed in biopsies from both sites and *in situ* zymography was used to measure and localize elastase-like activity in biopsy samples. Mucosal brushing samples from the same participants were also collected and total RNA purified for metatranscriptomic analysis to characterize active microbial pathways with a focus on identifying highly expressed microbial proteases.

**Results:**

Paracellular permeability and tissue conductance were greater in biopsies from inflamed areas than in biopsies from matched non-inflamed areas (p<0.001). Elastase-like and mucolytic activities were higher in inflamed areas compared to matched non-inflamed areas (*p*<0.05 and p=0.04, respectively), no significance was detected in total proteolytic activity. *In situ* zymography revealed greater elastase-like activity in inflamed areas compared to non-inflamed areas (p=0.04), localized to both the epithelial and submucosa layers in inflamed tissues but only to the epithelial layer in matched non-inflamed tissue. Preliminary analysis of the metatranscriptomics results suggests that specific microbial proteases may be increased at inflamed sites in CD.

**Conclusions:**

Our results suggest that localised variations in tissue inflammation, PA and mucosal permeability in CD may be causally linked, providing a rationale for the patchy distribution of mucosal injury. We, therefore, hypothesize that microbial elastase-like activity promotes mucosal inflammation through barrier disruption. This mechanism could be targeted locally to prevent or modulate microbially associated PA with the aim of enhancing mucosal healing in IBD.

Funding Agencies:

